# Overexpression of Phosphomimic Mutated OsWRKY53 Leads to Enhanced Blast Resistance in Rice

**DOI:** 10.1371/journal.pone.0098737

**Published:** 2014-06-03

**Authors:** Tetsuya Chujo, Koji Miyamoto, Satoshi Ogawa, Yuka Masuda, Takafumi Shimizu, Mitsuko Kishi-Kaboshi, Akira Takahashi, Yoko Nishizawa, Eiichi Minami, Hideaki Nojiri, Hisakazu Yamane, Kazunori Okada

**Affiliations:** 1 Biotechnology Research Center, The University of Tokyo, Bunkyo-ku, Tokyo, Japan; 2 Genetically Modified Organism Research Center, National Institute of Agrobiological Sciences, Tsukuba, Ibaraki, Japan; 3 Department of Biosciences, Teikyo University, Utsunomiya, Tochigi, Japan; China Agricultural University, China

## Abstract

WRKY transcription factors and mitogen-activated protein kinase (MAPK) cascades have been shown to play pivotal roles in the regulation of plant defense responses. We previously reported that *OsWRKY53*-overexpressing rice plants showed enhanced resistance to the rice blast fungus. In this study, we identified OsWRKY53 as a substrate of OsMPK3/OsMPK6, components of a fungal PAMP-responsive MAPK cascade in rice, and analyzed the effect of OsWRKY53 phosphorylation on the regulation of basal defense responses to a virulence race of rice blast fungus *Magnaporthe oryzae* strain Ina86-137. An *in vitro* phosphorylation assay revealed that the OsMPK3/OsMPK6 activated by OsMKK4 phosphorylated OsWRKY53 recombinant protein at its multiple clustered serine-proline residues (SP cluster). When OsWRKY53 was coexpressed with a constitutively active mutant of OsMKK4 in a transient reporter gene assay, the enhanced transactivation activity of OsWRKY53 was found to be dependent on phosphorylation of the SP cluster. Transgenic rice plants overexpressing a phospho-mimic mutant of OsWRKY53 (OsWRKY53SD) showed further-enhanced disease resistance to the blast fungus compared to native *OsWRKY53*-overexpressing rice plants, and a substantial number of defense-related genes, including pathogenesis-related protein genes, were more upregulated in the *OsWRKY53SD*-overexpressing plants compared to the *OsWRKY53*-overexpressing plants. These results strongly suggest that the OsMKK4-OsMPK3/OsMPK6 cascade regulates transactivation activity of OsWRKY53, and overexpression of the phospho-mimic mutant of OsWRKY53 results in a major change to the rice transcriptome at steady state that leads to activation of a defense response against the blast fungus in rice plants.

## Introduction

Plants protect themselves against pathogens through an innate immune system comprised of two layers [1]–[4]. The first layer is pathogen-associated molecular pattern (PAMP)-triggered immunity (PTI), activated by the recognition of PAMPs (e.g. bacterial flagellin and fungal chitin oligosaccharide) via plant pattern-recognition receptors. To prevent the colonization of plant tissue by pathogens, plants activate basal defense responses, such as synthesis of pathogenesis-related (PR) proteins and accumulation of phytoalexins, after perception of PAMPs by plant receptors. The second layer is effector-triggered immunity (ETI), which is a more accelerated defense response than PTI and is triggered by host-resistance (R) protein-mediated recognition of pathogen effectors. Activation of mitogen-activated protein kinases (MAPKs) and transcriptional regulation of defense-related gene expression are central to the induction of disease resistance in higher plants, and a number of transcription factor families (e.g. WRKY) have been identified [5] as associated with plant defense responses.

MAPK cascades are signaling systems that are evolutionally conserved among eukaryotes [6]. A basic MAPK cascade consists of 3 interconnected kinases: a MAPK, a MAPK kinase (MAPKK), and a MAPKK kinase (MAPKKK). In tobacco, 2 MAPKs, a wound-induced protein kinase (WIPK) and a salicylic acid-induced protein kinase (SIPK) are involved in both PAMP-triggered basal defense responses against fungal pathogens and ETI [7]–[12]. These 2 MAPKs share a common upstream MAPKK (NtMEK2) and function together in a single MAPK cascade. In *Arabidopsis*, it has been shown that AtMPK3 and AtMPK6, homologues of WIPK and SIPK respectively, are activated by recognition of PAMPs. They also share common upstream MAPKKs, namely AtMKK4 and AtMKK5 [13], [14]. The AtMPK3/AtMPK6 cascade plays a role in activation of defense-related genes, generation of reactive oxygen species (ROS), hypersensitive response-like cell death, and production of an indole-derived phytoalexin, camalexin [14]–[18]. It has also been shown that rice WIPK and SIPK homologues, OsMPK3 and OsMPK6, are activated by a fungal chitin oligosaccharide elicitor via a MAPKK (OsMKK4) and have important roles in cell death, biosynthesis of diterpenoid phytoalexins and lignin accumulation [19].

WRKY proteins form a large family of plant-specific transcription factors, and there are 74 and 109 genes encoding WRKY proteins in the *Arabidopsis* and rice genomes respectively [20], [21]. In numerous plant species, transcription of WRKY genes is strongly and rapidly upregulated in response to pathogen infection or treatment with either PAMPs or defense-related plant hormones [22]. WRKY proteins recognize the W-box elements ([T/C]TGAC[C/T]) in promoter regions of pathogen- or PAMPs-responsive genes like those encoding PR proteins, and modulate host defense against various phytopathogens as either positive or negative regulators [5], [22]–[25]. WRKY proteins contain 1 or 2 almost invariant WRKY domains composed of the conserved WRKYGQK amino acid sequence at the N terminus followed by a zinc-finger motif (CX_4–7_CX_22–23_HXH/C). They are divided into 3 groups based on the number of WRKY domains present: 2 WRKY domains with a C_2_H_2_ zinc finger motif (group I); 1 WRKY domain with a C_2_H_2_ zinc finger motif (group II); and 1 WRKY domain with a C_2_H/C zinc finger motif (group III) [26]. In the N-terminal region of several group I WRKY proteins, multiple clustered serine-proline residues (SP cluster), which can be putatively phosphorylated by MAPKs, are highly conserved (Fig. S1) [27]–[30].

Research to date has demonstrated that plant MAPK cascades regulate downstream gene expression through phosphorylation of group I WRKY proteins in defense-related signaling pathways. *Arabidopsis* AtWRKY33 exists in the nuclear complex with MAP KINASE 4 SUBSTRATE 1 (MKS1) and AtMPK4, and this ternary complex depends on MKS1. Complexes with MKS1 and AtWRKY33 are released after phosphorylation of MKS1 by the MAPK cascade AtMEKK1-AtMKK1/2-AtMPK4 in response to PAMP, and AtWRKY33 activates expression of *PAD3*, which is required for the synthesis of camalexin [31], [32]. It has also been demonstrated that AtWRKY33 is phosphorylated within the SP cluster by AtMPK3/AtMPK6, resulting in a stimulation effect on camalexin production [33]. In tobacco, SIPK phosphorylates NtWRKY1, resulting in enhanced DNA-binding activity of NtWRKY1 to the W-box, and co-expression of SIPK and NtWRKY1 enhanced SIPK-induced cell death [34]. In *Nicotiana benthamiana*, NbWRKY8 was identified as a substrate of SIPK, WIPK, and NTF4. These MAPKs phosphorylated NbWRKY8 within the SP cluster, and enhanced both DNA-binding and transactivation activities of NbWRKY8. Ectopic expression of the phospho-mimicking mutant of NbWRKY8 induced expression of defense-related genes, such as 3-hydroxy-3-methylglutaryl CoA reductase 2, that are involved in the production of isoprenoid phytoalexins in solanaceous plants [29]. In rice, OsWRKY33 is phosphorylated by OsBWMK1 which results in enhanced DNA-binding activity to the W-box elements [35].

Previously, we demonstrated that expression of *OsWRKY53*, one of the rice group I WRKY protein genes, was rapidly induced by either a fungal chitin oligosaccharide elicitor treatment or by infection with the blast fungus *Magnaporthe oryzae*, and 3 tandem W-box elements in the promoter of this gene were essential for the elicitor response. We also found that overexpression of *OsWRKY53* upregulated several defense-related genes in rice cells and resulted in enhanced resistance to a virulence race of *M. oryzae* in rice plants [36], [37]. OsWRKY53 has a conserved SP cluster in the N terminal region of the protein as found for other reported group I WRKY proteins (Fig. S1). Given that OsWRKY53 is the closest homologue of NbWRKY8 in rice [29], we hypothesized that OsWRKY53 is regulated by OsMPK3 and OsMPK6 at the posttranslational level, as part of a basal defense-signaling pathway in rice.

Here, we report that posttranslational regulation of OsWRKY53 plays an important role in regulating the basal defense response of rice plants against rice blast fungus through activation of the expression of defense-related genes. The OsMKK4-OsMPK3/OsMPK6, components of a fungal PAMP-responsive MAPK cascade in rice, phosphorylates the SP cluster of OsWRKY53 *in vitro*. OsMPK6-mediated phosphorylation of OsWRKY53 did not alter the DNA-binding activity to W-box elements, but co-expression of OsWRKY53 with a constitutively active OsMKK4 increased transactivation activity in an SP cluster-dependent manner. Transgenic rice plants overexpressing a phospho-mimic mutant of *OsWRKY53* leads to enhancement of disease resistance to a virulence race of rice blast fungus compared to native *OsWRKY53*-overexpressing rice plants. In addition, transcriptome analysis revealed that substantial numbers of defense-related genes were upregulated in the phospho-mimic mutant of *OsWRKY53*-overexpressing rice plants without blast fungus infection.

## Materials and Methods

### Plants, chemical treatment, pathogen, and rice transformation

Suspension-cultured rice cells (*Oryza sativa* L. cv. ‘Nipponbare’) were maintained as described previously [36]. Six days after subculturing, a small portion of the rice cells was harvested for particle bombardment. Rice plants (*Oryza sativa* L. cv. ‘Nipponbare’) were used in this study. Rice plants were grown in a chamber following previously described protocols [38]. The blast fungus *M. oryzae* strain Ina86–137 (MAFF 101511, race, 007.0, virulent to Nipponbare) was used for infection, and water was adopted as a mock treatment in this study. Rice transformation was performed as described previously [39].

### Plasmid construction

Plasmids containing OsWRKY53 SP cluster DNA fragments with alanine or aspartic acid substitutions at 6 serine residues in the cluster were generated by Takara Bio Inc. (Takara Bio Inc., Japan), resulting in pW53SA and pW53SD respectively.

To construct mutated *OsWRKY53* genes in which all 6 serine residues in SP cluster were substituted for alanine (*OsWRKY53SA*) or aspartic acid (*OsWRKY53SD*), the mutated SP cluster region was amplified from pW53SA or pW53SD by PCR using the primers W53-Ala F and W53-Ala R, or W53-Asp F and W53-Asp R, respectively. The amplified mutated SP cluster DNA fragments were used as reverse primers for PCR with forward primer W53-N Fw or 53 GAL4 F, respectively, to amplify *OsWRKY53* N-terminal regions containing the mutated SP cluster from pET-W53. After the second PCR, the amplified DNA fragments were used as forward primers for PCR with reverse primer W53-C Rv or 53 GAL4 R, respectively, to amplify whole *OsWRKY53SA* ORF or *OsWRKY53SD* ORF from pET-W53. The amplified DNA fragments were directly cloned into the pZErO2 vector (Invitrogen, CA, USA) and sequenced, resulting in pZE-W53SA or pZE-W53SD, respectively.

To construct the thioredoxin-6× histidine tag (Trx-His)-fused *OsWRKY53SA* gene, the *OsWRKY53SA* ORF was excised from pZE-W53SA by *Eco*RV and *Hin*dIII digestion and was inserted between corresponding sites of pET-32b(+) (Novagen, Germany) to generate pET-W53SA. To construct the Trx-His-fused *OsWRKY53SD* gene, the *OsWRKY53SD* ORF was amplified from pZE-W53SD by PCR using the primers W53-N and W53-R. The amplified DNA fragment was directly cloned into the pZErO2 vector (Invitrogen) to generate pZE-W53SD2. After performing a sequence check, the DNA fragment was excised from pZE-W53SD2 by *Eco*RV and *Hin*dIII digestion and was inserted between corresponding sites of pET-32b(+) to generate pET-W53SD.

To construct the plasmids containing *OsWRKY53* promoter region with W-box or mutated W-box elements, the corresponding promoter regions were amplified by PCR using the primers W53 Wbox Fw and W53 Wbox Rv from pZE-W53P2.0 or pZE-W53PmG, respectively. The amplified DNA fragments were directly cloned into a pT7Blue T-vector (Novagen) and sequenced, resulting in pT7-W53PW and pT7-W53mPW.

To construct the DNA-binding domain of the yeast transcription factor GAL4 (GAL4DB)-fused *OsWRKY53SA* gene, the *OsWRKY53SA* ORF was amplified from pZE-W53SA by PCR using the primers 53 GAL4 F and 53 GAL4 R. The amplified DNA fragment was directly cloned into the pZErO2 vector (Invitrogen) to generate pZE-W53SA2. After performing a sequence check, the DNA fragment was excised from pZE-W53SA2 by *Sma*I and *Sal*I digestion and was inserted between corresponding sites of 430T1.2 [40] to generate 35S-GAL4DB-W53SA. To construct GAL4DB-fused *OsWRKY53SD* gene, the *OsWRKY53SD* ORF was excised from pZE-W53SD by *Sma*I and *Sal*I digestion and inserted between corresponding sites of 430T1.2 [40] to generate 35S-GAL4DB-W53SD.

To construct Gateway destination vectors containing *Zea mays* polyubiquitin promoter and *Agrobacterium tumefaciens* nopaline synthase terminator, pUCAP/Ubi-NT [41] was digested with *Bam*HI and *Sac*I and blunt-ended. Then, reading frame cassette A (RfA) (Invitrogen) containing *att*R recombination sites flanking a *ccd*B gene and a chloramphenicol-resistance gene was cloned into blunt-ended pUCAP/Ubi-NT, resulting in pUbi_RfA_Tnos.

To construct effector plasmids in which GUS and a constitutively active form of OsMKK4, OsMKK4^DD^, were under the control of the *Z. mays polyubiquitin* promoter, GUS and OsMKK4^DD^ were cloned into pUbi_RfA_Tnos from pENTR-GUS (Invitrogen) and pENTR-MKK4^DD^ using LR clonase II Enzyme mix (Invitrogen). The resultant plasmids were designated as pUbi_GUS_Tnos and pUbi_MKK4^DD^_Tnos, respectively.

To construct a Gateway entry clone containing *OsWRKY53SD* ORF, *OsWRKY53SD* ORF was amplified by PCR using the primers 53 ORF Gateway F and OsWRKY53 pENTR R from 35S-GAL4DB-W53SD. The amplified DNA fragment was cloned into pENTR/D-TOPO (Invitrogen) according to manufacturer's protocol and sequenced, resulting in pENTR-W53SD.

To construct a binary vector in which *OsWRKY53* ORF and *OsWRKY53SD* ORF were under the control of the *Z. mays polyubiquitin* promoter for rice transformation, *OsWRKY53* ORF and *OsWRKY53SD* ORF were cloned into p2KG [42] from pENTR-W53 and pENTR-W53SD using LR clonase II Enzyme mix (Invitrogen). The resultant plasmids were designated as p2KG-W53 and p2KG-W53SD. A summary of the plasmids used in this study is provided in Table S1, and sequences of PCR primers used for plasmid construction are provided in Table S2.

### Recombinant proteins, *in vitro* kinase assays, and immunoblot analysis

OsMKK4^DD^ and OsMPK3/6 were expressed as fusion proteins with an N-terminus poly-histidine tag using a bacterial expression system (Invitrogen) following the manufacturer's instructions [19]. OsWRKY53 variants were also expressed as fusion proteins with an N-terminus poly-histidine tag as described previously [36]. The proteins were purified by immobilized metal ion affinity chromatography. To detect the phosphorylation of OsWRKY53 variants by recombinant OsMPKs, 800 ng of each recombinant OsMPK was pre-incubated for 1 h at 25°C with 120 ng of OsMKK4^DD^ in phosphorylation buffer containing 20 mM HEPES-KOH (pH 7.5), 10 mM MgCl_2_, 1 mM DTT, and 50 µM ATP, and then was incubated with 1.2 µg of each OsWRKY53 variant and 25 µM ATP for 1 h at 25°C. The reaction was stopped by the addition of Laemmli's SDS sample buffer and boiling. The above samples were subjected to SDS-PAGE on 10% (w/v) polyacrylamide gels, transferred to Immobilon-P Transfer Membrane (PVDF, 0.45 µm) (Millipore, MA, USA). Phosphorylated OsWRKY53 were detected by using Phos-tag Biotin BTL-104 (Wako, Japan) according to the manufacturer's instruction. OsWRKY53 variants, OsMPK3 and OsMPK6, were detected with anti-His antibody [dilution 1∶3000 (v/v)] (GE Healthcare, UK), anti-OsMPK3 serum [dilution 1∶3000 (v/v)] and anti-OsMPK6 serum [dilution 1∶5000 (v/v)], respectively, as the primary antibody, and ECL anti-mouse IgG horseradish peroxidase-linked species-specific whole antibody (dilution 1∶25,000 [v/v]) (GE Healthcare) and ECL anti-rabbit IgG horseradish peroxidase-linked species-specific whole antibody (dilution 1∶25,000 [v/v]) (GE Healthcare) as the secondary antibody. Chemiluminescent detection was carried out with the Immobilon Western Chemiluminescent HRP Substrate (Millipore) according to the manufacturer's instruction.

### Gel mobility shift assays

Double-stranded W-box or mutated W-box probes were amplified by PCR from pT7-W53PW or pT7-W53mPW using the primers W53 Wbox Fw and W53 Wbox Rv and end-labeled with ^32^P by T4 polynucleotide kinase (Takara Bio). The probe was purified using Illustra Microspin G-25 columns (GE Healthcare). Phosphorylation of OsWRKY53 recombinant protein was described above in the “Recombinant proteins, in vitro kinase assays, and immunoblot analysis” section. The gel mobility shift assay (GMSA) reaction mixture comprised 12 mM HEPES-KOH (pH 8.0), 60 mM KCl, 4 mM MgCl_2_, 1 mM EDTA, 12% glycerol, 1 mM DTT, 2.5 mM PMSF, 0.1 µg of recombinant OsWRKY53, and 2 µl of the probe in a final volume of 20 µl. To form DNA-protein complexes, the above mixed samples were incubated for 20 min on ice. Finally, samples were separated on a 6% polyacrylamide gel in 1× TBE at room temperature, and bands were visualized by autoradiography.

### Transactivation assay

pUbi_GUS_Tnos and pUbi_MKK4^DD^_Tnos, in which GUS and OsMKK4^DD^, respectively, are under the control of the *Z. mays polyubiquitin* promoter, were used as the OsMKK4-variants effector plasmids. 430T1.2, 35S-GAL4DB-W53, 35S-GAL4DB-W53SA, and 35S-GAL4DB-W53SD, in which GAL4DB and GAL4DB-OsWRKY53 variants are under the control of the CaMV *35S* promoter, were used as the GAL4DB-OsWRKY53-variants effector plasmids. GAL4-TATA-LUC-NOS, which contains a firefly luciferase (LUC) gene, was used as a reporter plasmid. The plasmid pRL, which contains the *Renilla* LUC gene under the control of the CaMV *35S* promoter, was used as an internal control. Particle bombardment was carried out with the PDS-1000 He Biolistic Particle Delivery System (Bio-Rad, CA, USA). Suspension-cultured rice cells were used for the bombardment. In co-transfection assays, 1.2 µg of the GAL4DB-OsWRKY53-variants effector construct, 1.6 µg of the reporter plasmid, and 0.4 µg of pRL with and without 1.2 µg of the OsMKK4-variants effector construct were used for each bombardment. Luciferase assays were performed with the Dual-Luciferase Reporter Assay System (Promega, WI, USA) and a Centro LB960 plate reader (Berthold Japan, Japan) following the manufacturer's instructions. The ratio of LUC activity (firefly LUC/*Renilla* LUC) was calculated to normalize values after each assay.

### Quantitative RT-PCR

Total RNA was extracted from rice leaves of non-transformed control (NT), *OsWRKY53-overexpressing* (*W53-OX*), *OsWRKY53SD-overexpressing* (*W53SD-OX*) rice plants, and NT rice calli using an RNeasy Plant Mini Kit (Qiagen, Germany) and subjected to cDNA synthesis using a PrimeScript RT reagent Kit with gDNA Eraser (Takara Bio Inc.). Quantitative RT-PCR (qRT-PCR) was performed using TaqMan probe with THUNDERBIRD Probe qPCR Mix (TOYOBO, Japan) for *OsKSL4* and a Power SYBR Green PCR Master Mix (Applied Biosystems, CA, USA) for the other target genes on an ABI PRISM 7300 Real-Time PCR System (Applied Biosystems). To calculate the transcript levels of characterized genes, the copy number of their mRNAs was determined by generating standard curves using a series of known concentrations of the target sequence. Ubiquitin domain-containing protein (UBQ, Os10g0542200) was used as an internal control to normalize the amount of mRNA. For each sample, the mean value from triplicate amplifications was used to calculate the transcript abundance. Sequences of PCR primers and TaqMan probe used for qRT-PCR analysis are provided in Table S3.

### Pathogen inoculation and disease-resistance test

To examine fungal lesions and biomass, fungal inoculation of rice leaf blades was carried out as described previously [38] with some modifications. Fourteen-day-old rice plants were placed on moistened filter paper in plastic trays. Washed conidia of the blast fungus were suspended at a concentration of 1×10^5^ cells mL^−1^ in distilled water for the disease-resistance test, and were sprayed on rice plants incubated at 25°C in the dark for 24 h, followed by 14 h-light/10 h-dark cycles for 5 days. Lesions on the fourth leaves were classified as described previously [43]: necrotic spots (resistant dark brown specks), intermediate lesions (yellow and brown lesions without a gray center), and susceptible lesions (areas with a gray center and emerging aerial hyphae and conidia). Blast disease development was quantified by measurement of *M. oryzae* genomic DNA (encoding 28S rRNA) relative to rice genomic DNA (encoding the *eEF-1α* gene) using quantitative genomic PCR analysis [44]. Quantitative genomic PCR was performed using a SYBR Premix Ex Taq II (Takara Bio Inc.) on an MX3000P (Stratagene, CA, USA). Sequences of PCR primers used for qPCR analysis are provided in Table S4. Data are presented relative to the value in leaves of NT rice plants.

### Microarray data acquisition and cluster analysis

Fourth leaves of NT, *W53-OX*, and *W53SD-OX* rice plants were harvested from 4 plants at the 4-leaf stage. Total RNA was isolated from the rice leaves using an RNeasy Plant Mini Kit (Qiagen) and subjected to fluorescence labeling according to the manufacturer's instructions. The RNA was labeled with a Cyanine 3 dye (Cy3). Aliquots of Cy3-labeled cRNAs (1650 ng each) were used for hybridization in a 60-mer rice oligo microarray with 44k features (Agilent Technologies, CA, USA). Four biological replicate sample sets were analyzed. The glass slides were scanned using a microarray scanner (G2565, Agilent), and resulting output files were imported into Feature Extraction software (ver. 11; Agilent). Data normalization and statistical analyses were performed using Partek Genomics Suite software (ver. 6.5; Partek software, MO, USA). Data from the selected spots were imported into MultiExperiment Viewer (MeV v4.8, http://www.tm4.org/mev/) for cluster analysis. A hierarchical clustering analysis based upon the average linkage and cosine correlation was then used to cluster genes on the *y*-axis using MeV.

### Phytoalexin measurements

Rice leaves (15–50 mg FW) were soaked in 2 mL of phytoalexin extraction solvent (ethanol/water/acetonitrile/acetic acid, 79∶13.99∶7∶0.01, v/v) and heated for 15 min twice in a glass tube with a screw cap. The extract was centrifuged (4°C, 15 min, 16,000×*g*). The supernatant was collected and subjected to phytoalexin measurements by LC-ESI-MS/MS as described previously [45].

## Results

### *In vitro* phosphorylation of OsWRKY53 by OsMPK3 and OsMPK6

**Figure 1:**
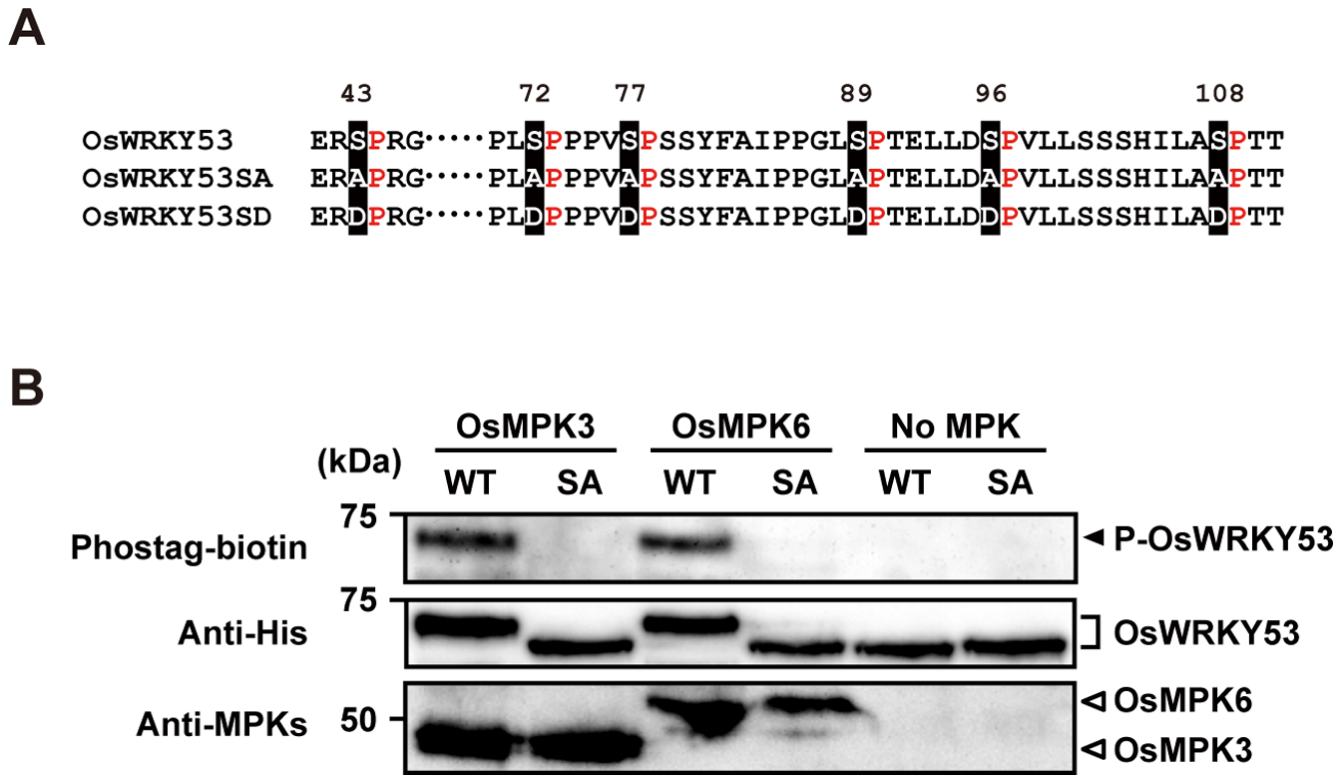
*In vitro* phosphorylation of OsWRKY53 by chitin-responsive OsMPKs. **A**, Putative MAPK phosphorylation sites in the SP cluster region of OsWRKY53, the loss-of-phosphorylation OsWRKY53 mutant with all 6 Ser substituted to Ala (OsWRKY53SA), and the phospho-mimicking OsWRKY53 mutant with all 6 Ser substituted to Asp (OsWRKY53SD). **B**, *In vitro* phosphorylation of OsWRKY53 by OsMPK3 and OsMPK6. Recombinant His-OsWRKY53 and His-OsWRKY53SA proteins were used as the substrate for rice mitogen-activated protein kinases (OsMPKs) activated by a constitutively active form of the rice MAP kinase OsMKK4^DD^. Proteins separated by SDS-PAGE were blotted on membrane and probed with a Phostag-biotin antibody (top panel). The arrowhead indicates the position of phosphorylated OsWRKY53 (P-OsWRKY53). The membranes were reprobed followed by probing with an anti-His antibody to detect added substrate OsWRKY53 and OsWRKY53SA proteins (middle panel). OsMPKs in the reaction mixtures were also detected by immunoblot analysis with anti-OsMPK3 and anti-OsMPK6 antiserum (bottom panel). WT, native His-OsWRKY53; SA, His-OsWRKY53SA.

First, we focused on potential MAPK phosphorylation sites of OsWRKY53, especially Ser or Thr residues followed by Pro, which is a minimal consensus motif for MAPK phosphorylation [27], [28]. The deduced amino acid sequence of OsWRKY53 possessed 6 SP and 2 TP motifs, 6 of which were concentrated in the N-terminal region as the SP cluster conserved among several group I WRKY proteins (Fig. 1A and Fig. S1). To investigate whether OsWRKY53 was phosphorylated by rice MAPKs, OsMPK3, OsMPK6 and a Thioredoxin-His-tagged OsWRKY53 recombinant proteins were expressed and purified from *E. coli*, and subjected to *in vitro* MAPK phosphorylation assay. As shown in Fig. 1B OsWRKY53 was phosphorylated by recombinant OsMPK3 and OsMPK6 activated by a constitutively active form of OsMKK4, OsMKK4^DD^. We also confirmed this phosphorylation did not occur without OsMPK3 and OsMPK6.

As the SP cluster of OsWRKY53 was hypothesized to be essential for the phosphorylation by OsMPK3 and OsMPK6, we prepared a Thioredoxin-His-tagged OsWRKY53SA recombinant protein in which all 6 Ser residues in the SP cluster were substituted for Ala (Fig. 1A), and performed an *in vitro* MAPK phosphorylation assay. Compared to the results of the original experiment using native OsWRKY53 recombinant protein, activated recombinant OsMPK3 and OsMPK6 could no longer phosphorylate OsWRKY53SA (Fig. 1B). In addition, we detected single shifted band of native OsWRKY53 by using anti-His antibody in an OsMPK3/OsMPK6 dependent manner (Fig. 1B). Given that this band shift is correlated with the phosphorylation of native OsWRKY53 by OsMPK3/OsMPK6, it is suggested that nearly all native OsWRKY53 protein molecules are phosphorylated in our experimental condition. Taken together, these results indicate that OsMPK3 and OsMPK6 can phosphorylate OsWRKY53 efficiently, and that phosphorylation site of OsWRKY53 by OsMPK3 and OsMPK6 resides in the SP cluster in the N-terminal region.

### Phosphorylation of OsWRKY53 by OsMPK6 does not alter its W-box binding activity

**Figure 2:**
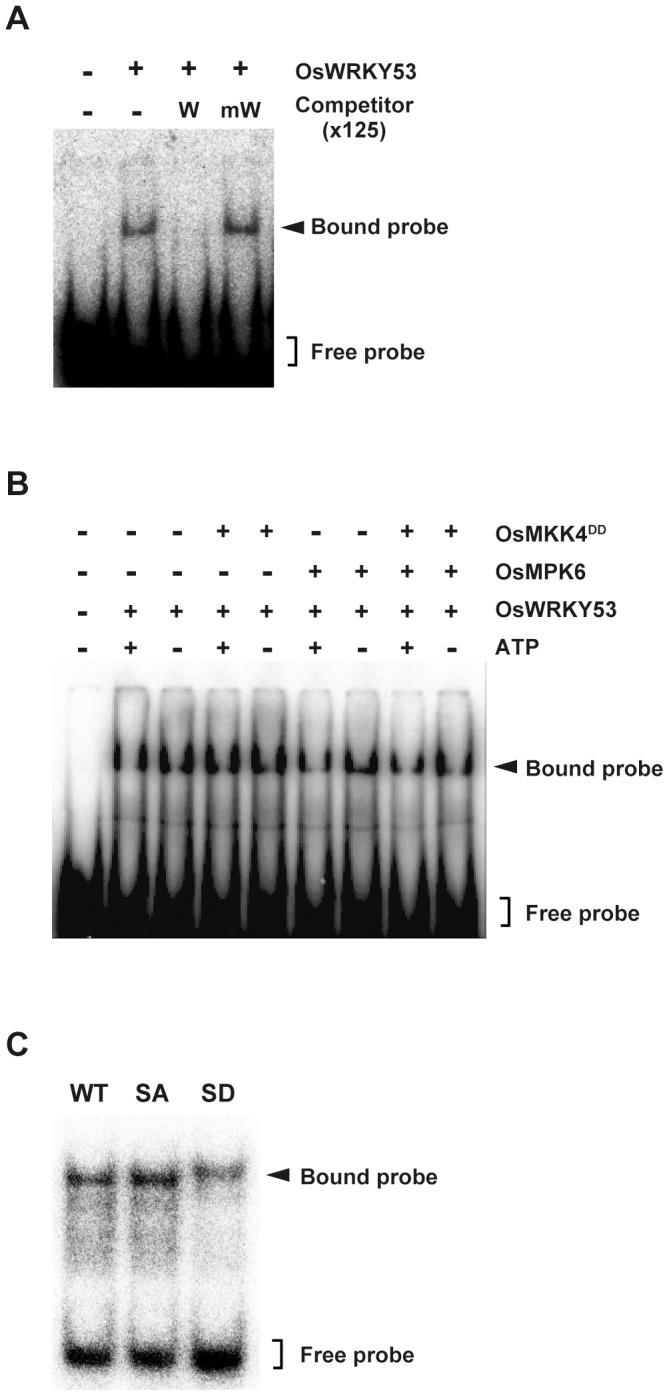
Phosphorylation of OsWRKY53 does not alter its W-box binding ability. **A**, W-box-specific DNA-binding activity of OsWRKY53. GMSA assay was performed using purified recombinant OsWRKY53 protein and ^32^P-labeled W-box probe containing the W-box *cis*-elements in the *OsWRKY53* promoter. The specificity of the W-box binding activity was demonstrated by competition assay using 125-fold excess amount of unlabeled W-box probe (W) and mutated W-box probe (mW). **B**, Phosphorylation of OsWRKY53 does not enhance its W-box binding activity. Purified recombinant OsWRKY53 protein was phosphorylated using the OsMPK6 activated by OsMKK4^DD^. GMSA was performed as in **A**. **C**, W-box binding activity of OsWRKY53 variant proteins. Purified recombinant OsWRKY53, OsWRKY53SA and OsWRKY53SD proteins were subjected to GMSA. GMSA was performed as in **A**. WT, native His-OsWRKY53; SA, His-OsWRKY53SA; SD, His-OsWRKY53SD.

It has been reported that phosphorylation of the group I WRKY proteins NtWRKY1 and NbWRKY8 by MAP kinase enhanced their W-box specific DNA-binding activity [29], [34]. To investigate whether phosphorylation of OsWRKY53 by OsMPK3/OsMPK6 enhanced W-box binding activity, we performed a gel mobility shift assay (GMSA). First, we analyzed the W-box-specific DNA binding activity of OsWRKY53 by using a W-box probe derived from the native promoter region of this gene. When incubated with the OsWRKY53 recombinant protein, a retarded band was observed, and this retardation of the labeled probe was abolished by competition using an unlabeled probe. In contrast, an unlabeled W-box mutated probe could not compete with the labeled probe (Fig. 2A).

The effect of the phosphorylation of OsWRKY53 by the rice MAP kinases on the W-box-specific DNA binding activity of OsWRKY53 was also tested by GMSA with phosphorylated OsWRKY53 protein. Because OsMPK3 and OsMPK6 showed approximately the same phosphorylation pattern for OsWRKY53, we used OsMPK6 to phosphorylate OsWRKY53 in this experiment. Recombinant OsWRKY53 protein was incubated with OsMPK6 alone, OsMKK4^DD^ alone, or OsMPK6 activated by OsMKK4^DD^, and subjected to GMSA. As a result, there is no distinct difference in the W-box binding activity between the phosphorylated OsWRKY53 and native OsWRKY53 (Fig. 2B) We performed further GMSA analysis using native OsWRKY53, OsWRKY53SA and a phospho-mimic mutant of OsWRKY53 in which all 6 Ser residues in the SP cluster were substituted for Asp (OsWRKY53SD) (Fig. 1A). As shown in Fig. 2C, these three WRKY53 variants had similar W-box binding activity. Taken together, these results suggest that activated OsMPK6-mediated phosphorylation of OsWRKY53 did not affect its DNA binding activity.

### OsMKK4^DD^ enhances transactivation activity of OsWRKY53

**Figure 3:**
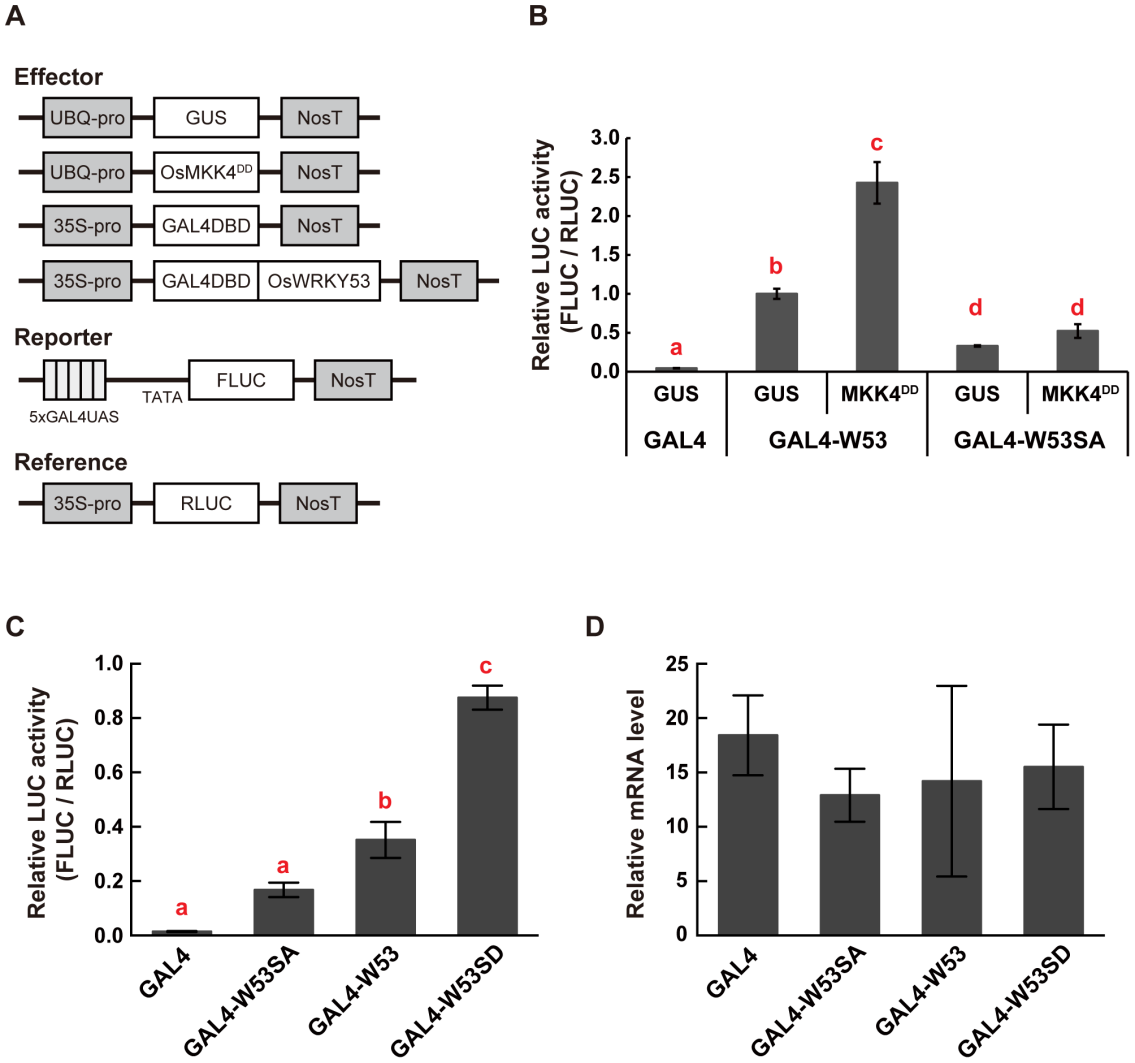
Post-translational regulation of OsWRKY53 transactivation activity by OsMKK4^DD^. **A**, Diagrams of effector, reporter, and reference plasmids used in transient reporter gene assays. **B**, Regulation of transactivation activity of OsWRKY53 by OsMKK4^DD^ in cultured rice cells. A GUS construct was used as a negative control. Firefly LUC activity was normalized against that of *Renilla* LUC. Values of LUC activity are shown relative to those of GAL4-W53 + GUS (n = 4); *bars* indicate the standard error of the mean. Three independent experiments were performed, and a representative result is shown. Statistically different data groups are indicated using different letters (*p*<0.01 by One-way ANOVA with Tukey post hoc test). **C**, Enhanced transactivation of a phosphorylation-mimic mutant of OsWRKY53 in cultured rice cells. Transient reporter gene assay was performed as in **B**. Values of LUC activity are shown relative to those of GAL4-W53 (n = 4); *bars* indicate the standard error of the mean. Three independent experiments were performed, and a representative result is shown. Statistically different data groups are indicated using different letters (*p*<0.05 by One-way ANOVA with Tukey post hoc test). **D**, Expression analysis of *GAL4DB* and *GAL4DB-OsWRKY53* variants in cultured rice cells. qRT-PCR analysis was performed using total RNA isolated from rice cells after particle bombardment with effector, reporter and reference plasmids. Values indicate relative mRNA levels normalized to the expression of the *RLUC* gene (n = 4); *bars* indicate the standard error of the mean. W53, W53SA and W53SD indicate the native OsWRKY53, an OsWRKY53 mutants whose Ser residues in the SP cluster were substituted to Ala, and an OsWRKY53 mutant that mimics the phosphorylated form, respectively.

It has also been reported that *N. benthamiana* MEK2^DD^ enhanced transactivation activity of NbWRKY8 [29]. To investigate whether the rice OsMKK4^DD^ correspondingly enhances transactivation activity of OsWRKY53, we performed a transient reporter gene assay. We constructed effector plasmids that contained a *GUS* or *OsMKK4^DD^* gene under the control of the maize *ubiquitin* promoter (Fig. 3A). We also constructed effector plasmids that contained the CaMV *35S* promoter, driving a gene that encodes a fusion protein of the DNA-binding domain of the yeast transcriptional activator GAL4 and the full-length OsWRKY53 or OsWRKY53SA (GAL4DB-OsWRKY53 or GAL4DB-OsWRKY53SA), and a control plasmid encoding only GAL4DB (Fig. 3A). Each of the GAL4DB-OsWRKY53-variant effector plasmids or the control plasmid was delivered into rice cells along with a reporter plasmid *GAL4-TATA-LUC-NOS*, which contained 5 tandem repeats of a GAL4 binding site fused to the firefly *LUC* (*FLUC*), and either *GUS* or *OsMKK4^DD^* effector plasmid by particle bombardment. As described in our previous report [36], coexpression of GAL4DB-OsWRKY53 with GUS showed more than 20-fold greater LUC activity compared to coexpression of GAL4DB with GUS as the negative control. On the other hand, LUC activity of rice cells coexpressing GAL4DB-OsWRKY53SA with GUS was reduced by 67% compared to those co-expressing GAL4DB-OsWRKY53 with GUS (Fig. 3B). Interestingly, coexpression of GAL4DB-OsWRKY53 with OsMKK4^DD^ significantly increased (more than doubled) the LUC activity (Fig. 3B). In contrast, coexpression of GAL4DB-OsWRKY53SA with OsMKK4^DD^ did not increase LUC activity (Fig. 3B). These results indicate that OsMKK4^DD^ can increase transactivation activity of OsWRKY53 in an SP cluster-dependent manner.

Given that OsWRKY53 is phosphorylated within the SP cluster by OsMPK3 and OsMPK6 activated by OsMKK4^DD^ *in vitro* (Fig. 1B), we expected that a phospho-mimic mutant of OsWRKY53 would show enhanced transactivation activity. To verify this hypothesis, we constructed an effector plasmid that encodes a fusion protein of the GAL4DB and OsWRKY53SD (GAL4DB-OsWRKY53SD) (Fig. 3A). Rice cells expressing GAL4DB-OsWRKY53SD showed more than 2-fold greater LUC activity compared to those coexpressing GAL4DB-OsWRKY53 (Fig. 3C). We also confirmed almost equal expression of *GAL4DB* and *GAL4DB-OSWRKY53* variants in these experiments by quantitative RT-PCR (qRT-PCR) analysis (Fig. 3D). Thus, these results strongly suggest that activation of OsMPK3 and OsMPK6 by OsMKK4 is an important mechanism for regulating the transactivation activity of OsWRKY53 *in vivo* by phosphorylation of OsWRKY53 within the SP cluster.

### Overexpression of a phospho-mimic mutant of OsWRKY53 further enhances the basal defense against rice blast fungus

We previously showed that *OsWRKY53*-overexpressing transgenic rice plants exhibited enhanced resistance to a virulence race of *M. oryzae* [36]. Given the result here that a phospho-mimic mutant of OsWRKY53 (OsWRKY53SD) shows enhanced transactivation activity compared to the native OsWRKY53, we hypothesized that overexpression of *OsWRKY53SD* would confer further enhanced disease resistance against the rice blast fungus in rice plants. To test this hypothesis, we generated transgenic rice plants that expressed either native *OsWRKY53* or *OsWRKY53SD* under the control of the constitutive maize *ubiquitin* promoter and tested the transformants for resistance to a virulence race of rice blast fungus, *M. oryzae* strain Ina86–137. The constructs for *OsWRKY53*- or *OsWRKY53SD*-overexpression were introduced into the rice cultivar Nipponbare by *Agrobacterium*-mediated transformation, and the overexpression of *OsWRKY53* or *OsWRKY53SD* in transgenic rice plants (*W53-OX* and *W53SD-OX*, respectively) was confirmed by qRT-PCR analysis (Fig. S2). Overexpression of *OsWRKY53* or *OsWRKY53SD* did not affect the growth and morphology of the transgenic rice plants (data not shown).

**Figure 4:**
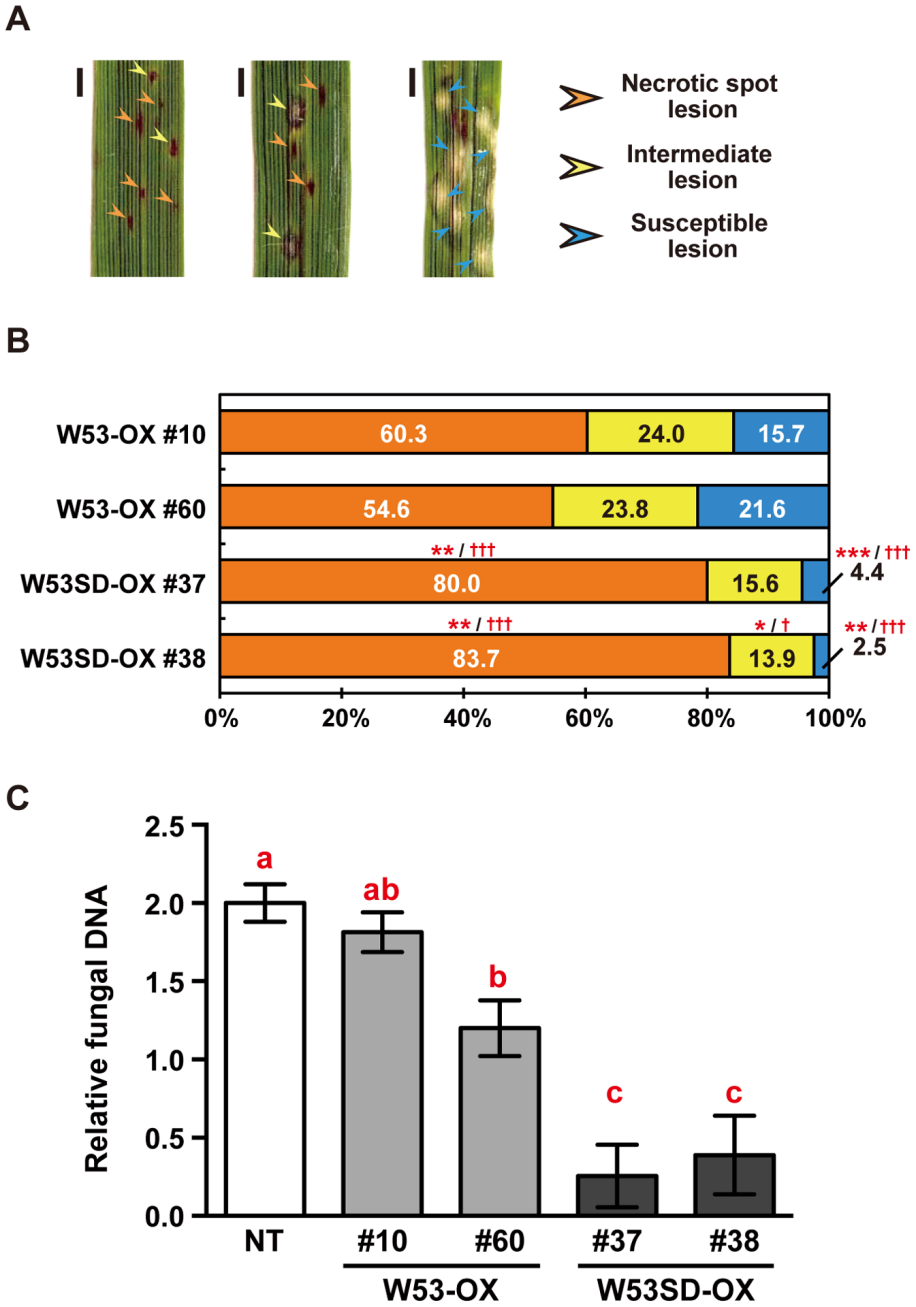
Overexpression of a phosphorylation-mimic mutant of *OsWRKY53* enhances the rice blast resistance of rice plants. **A**, Representative lesions of rice blast disease observed at 5 days post inoculation (dpi). Bars indicate 1 mm. **B**, Ratio of the classes of lesions in transgenic rice leaves infected with rice blast fungus *Magnaporthe oryzae* Ina86–137. Washed conidia of the blast fungus were suspended in 1 mM MES-NaOH (pH 5.7) and then were inoculated on leaves of *W53-OX* and *W53SD-OX* transgenic rice plants. The lesions were counted according to the classifications shown in **A**. Bars represent the ratio of lesions of each class to the total number of counted lesions in 3 or 4 individual leaf blades. Three independent experiments were performed, and a representative result is shown. *Asterisks* and *daggers* denote significant differences compared to *W53-OX* plants (*p<0.05; **p<0.01; ***p<0.001 vs. *W53-OX* #10; ^†^ *p*<0.05; ^†††^ *p*<0.001 vs. *W53-OX* #60, by One-way ANOVA with Tukey post hoc test) **C**, Development of blast disease in leaf blades evaluated by quantitating *M. oryzae* genomic DNA. The amount of *M. oryzae* 28S rDNA relative to rice genomic *eEF1α* DNA was determined by quantitative PCR analysis. Values are represented as mean values ±SE for 6 leaf blades. Statistically different data groups are indicated using different letters (*p*<0.05 by One-way ANOVA with Tukey post hoc test on log-transformed data). NT, non-transformant control rice plants; *W53-OX*, native *OsWRKY53*-overexpressing rice plants; *W53SD-OX*, phosphorylation-mimic mutant of *OsWRKY53*-overexpressing rice plants.

Next, we tested the transformants for resistance to the blast fungus. The transgenic rice plants were grown in a growth chamber, inoculated with conidia of the blast fungus, and disease symptoms were characterized 5 days later. We confirmed as previously reported that *W53-OX* rice plants showed enhanced resistance against the blast fungus compared to non-transformed control (NT) rice plants (Fig. S3). Thus, we compared resistance to the blast fungus between *W53-OX* and *W53SD-OX* rice plants directly. We did not see statistically-significant differences in average numbers of lesions in the total infected area between 2 independent *W53-OX* and 2 independent *W53SD-OX* samples (Fig. S4). Therefore, we categorized the disease lesions into 3 classes (Fig. 4A) and compared the ratio of lesions between *W53-OX* and *W53SD-OX* rice plants. Interestingly, the ratio of necrotic spot lesions in leaves of the *W53SD-OX* plants was significantly increased relative to that of *W53-OX* plants (Fig. 4B). Moreover, the ratio of susceptible lesions in *W53SD-OX* plants was significantly decreased relative to *W53-OX* plants (Fig. 4B). We also quantified fungal biomass in leaves of NT, *W53-OX* and *W53SD-OX* rice plants. Quantitative genomic PCR analysis demonstrated that significantly reduced levels of fungal DNA were detected from *W53SD-OX* plants compared with *W53-OX* plants. It was also indicated that fungal biomass levels tend to be decreased in *W53-OX* plants compared with NT plants (Fig. 4C). These results strongly suggest that overexpression of a phospho-mimic mutant of *OsWRKY53* further enhances the basal defense response against the virulence rice blast fungus compared to native *OsWRKY53*-overexpressing rice plants.

### Genome-wide profiling of gene expression in *W53-OX* and *W53SD-OX* rice plants

To examine the transcriptional changes that accompany the enhanced disease resistance in *W53SD-OX* rice plants, we performed a genome-wide DNA microarray analysis using NT, *W53-OX*, and *W53SD-OX* rice plants, and gene expression was compared between *W53-OX* and *W53SD-OX*. These data have been deposited in the Gene Expression Omnibus in NCBI (http://www.ncbi.nlm.nih.gov/geo/; ID:GSE48500). We performed 4 biological replicates with 2 independent *W53-OX* (#8 and #10) or *W53SD-OX* (#38 and #40) rice-plant lines. The statistical analysis was performed on normalized data using the ANOVA-false discovery rate (ANOVA-FDR, q value ≤0.05) as calculated by Partek Genomics Suite (http://www.partek.com/), and we selected genes with changes in expression based on the criterion of a twofold increase or decrease in the average levels of fold change in *W53SD-OX* relative to expression levels in *W53-OX*. Based on this criterion, 280 genes were upregulated and 135 genes were downregulated in *W53SD-OX* rice plants (Table S5).

Given that OsWRKY53 is a transactivator whose expression is upregulated upon blast infection [36], it is likely that its target genes involved in the blast resistance are also upregulated in NT rice plants upon blast fungus infection. Thus, we first focused on the 280 upregulated genes in *W53SD-OX* rice plants compared to *W53-OX* rice plants, and compared those with the results of our previous transcriptome analysis using NT rice plants with and without blast infection (http://www.ncbi.nlm.nih.gov/geo/; ID: GSE39635). This analysis showed that 151 out of the 280 upregulated genes in *W53SD-OX* rice plants were also upregulated in NT rice plants infected with the blast fungus. Next, the 151 genes that showed increased expression upon blast infection were subjected to hierarchical clustering using the Pearson correlation and average linkage methods together with the microarray data of NT rice plants. Based on their expression pattern in the rice plants, we classified the 151 upregulated genes into the following 2 major groups: group I, genes whose expression increased in *W53SD-OX* plants compared to NT, and increased a little or not in *W53-OX* plants; group II, genes whose expression decreased in both *W53-OX* and *W53SD-OX* plants compared to NT. This analysis showed that most of the genes (94%) were included in the group I, and more than 70% of the group I genes showed increased expression pattern in a stepwise fashion in *W53-OX* and *W53SD-OX* plants compared to NT (Fig. S5, Table S5).

### Overexpression of Os*WRKY53SD* enhances the activation of defense-related genes

**Figure 5:**
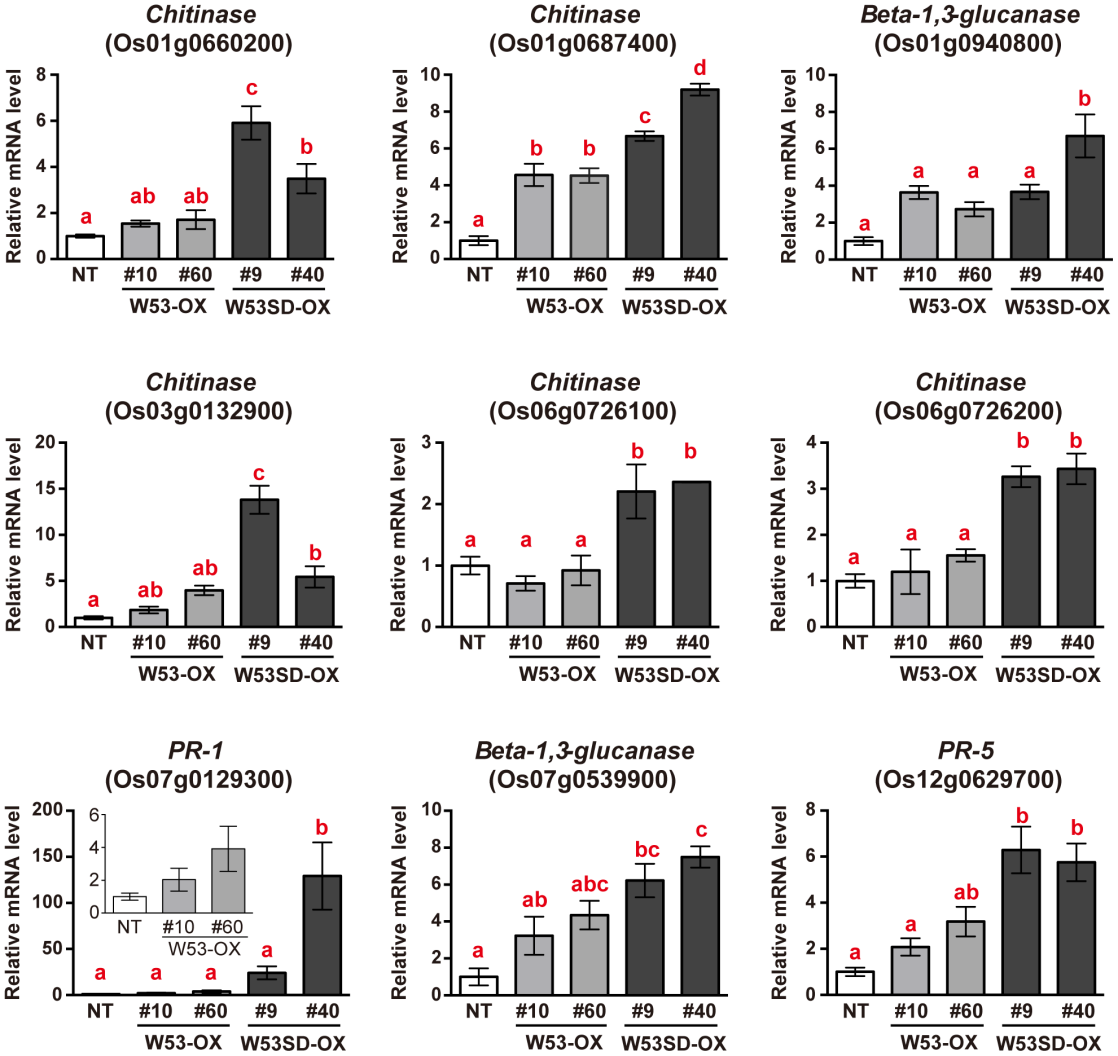
Expression analysis of defense-related genes belonging to group I upregulated genes in *W53SD-OX* rice plants. qRT-PCR analysis was performed using total RNA isolated from uninfected rice leaves. Values indicate relative mRNA levels normalized to the expression of the *UBQ* gene (n = 3); *bars* indicate the standard error of the mean. Three independent experiments were performed, and a representative result is shown. Statistically different data groups are indicated using different letters (*p*<0.05 by One-way ANOVA with Tukey post hoc test). NT, non-transformed control; OX, *OsWRKY5*3-overexpressing rice plants; SD-OX; *OsWRKY53SD*-overexpressing rice plants.

**Figure 6:**
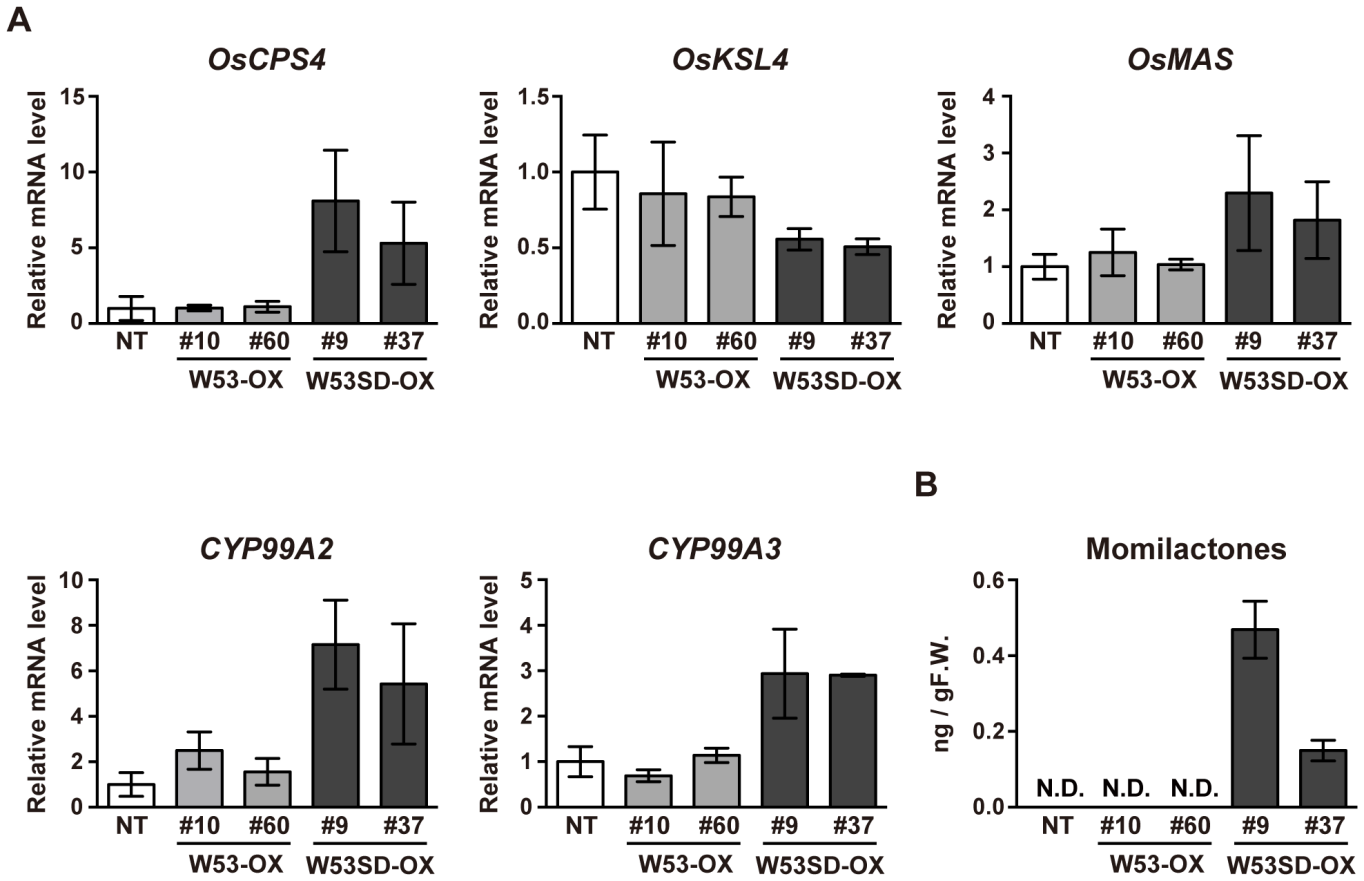
Phytoalexin accumulation in *W53SD-OX* rice plants. **A**, Expression analysis of momilactone biosynthetic genes. qRT-PCR analysis was performed using total RNA isolated from uninfected rice leaves. Values indicate relative mRNA levels normalized to the expression of the *UBQ* gene (n = 3); *bars* indicate the standard error of the mean. Three independent experiments were performed, and a representative result is shown. **B**, Accumulation of momilactones in uninfected *W53SD-OX* rice plants. Momilactone levels in the rice leaves were determined by LC-MS/MS. The results are the average of at least 3 independent experiments. *Bars* indicate the standard error of the mean. NT, non-transformed control; OX, *OsWRKY5*3-overexpressing rice plants; SD-OX; *OsWRKY53SD*-overexpressing rice plants. N.D.: not detected.

Based on the results described above, it appears that a subset of defense-related genes is further activated in *W53SD-OX* rice plants compared to *W53-OX* plants. Thus, the 151 genes upregulated in *W53SD-OX* were organized into molecular function Gene Ontology (GO) categories based on their primary functions. Most of the genes grouped into the following categories: binding (GO:0005488), catalytic activity (GO:0003824), nucleic acid binding transcription factor activity (GO:0001071), and transporter activity (GO:0005215). But some of these genes grouped into the following 2 non-GO categories: defense-related genes and molecular function unknown (Table S5). Besides, almost all of the defense-related genes (22 out of 23 genes) among the 151 upregulated genes fell into the group I, including several PR protein genes such as β-1,3-glucanase, chitinase, and *PR-5* genes (Table 1). Given that *W53SD-OX* rice plants showed further-enhanced resistance to the virulence rice blast fungus, we finally focused on the group I genes, especially those relevant to defense response. To further validate the results of the microarray analysis, we performed qRT-PCR analysis using NT and each of 2 independent *W53-OX* and *W53SD-OX* rice plants. As shown in Fig. 5, the expression of most of these genes was significantly upregulated in *W53SD-OX* plants compared to NT plants, and some of these genes' transcripts were also accumulated in *W53-OX* plants compared to NT. We also noticed that *OsCPS4* and *CYP99A2*, which encoded biosynthetic enzymes of rice diterpenoid phytoalexins (momilactones), were included in the above defense-related genes (Table 1). Momilactones are synthesized from geranylgeranyl diphosphate (GGDP) through 2 cyclization and multiple oxidation steps [46], [47]. We therefore examined the expression levels of these above 2 genes (*OsCPS4* and *CYP99A2*) and 3 additional genes (*OsKSL4*, *OsMAS* and *CYP99A3*) for momilactone biosynthesis by qRT-PCR analysis. We found that transcript levels of these momilactone-biosynthetic genes except *OsKSL4* were increased only in the *W53SD-OX* rice plants, a result consistent with the basal accumulation of momilactones in these plants (Fig. 6). Taken together, these results suggest that the potentiated induction of these defense-related genes at steady state provides further enhanced disease resistance of *W53SD-OX* rice plants against rice blast fungus at the time of infection compared to *W53-OX* rice plants.

**Table 1:** List of defense related genes belonging to Group I.

Locus ID	Accession Number	Description	Fold change (*W53SD-OX* vs *W53-OX*)	*q*-value
Os01g0660200	AK100973	Chitinase	2.69706	0.00050024
Os01g0687400	AK106178	Chitinase	2.14256	0.000193826
Os01g0713200	AK060113	Beta-1,3-glucanase	3.09387	0.000710591
Os01g0860500	AK103976	Chitinase	2.13807	0.0317721
Os01g0940700	AK070677	Beta-1,3-glucanase	2.6235	0.00416658
Os01g0940800	AK105972	Beta-1,3-glucanase	2.31721	0.0229786
Os01g0963000	AK102172	Peroxidase	2.19554	0.0167012
Os03g0132900	AK099355	Chitinase	2.88386	0.0029401
Os04g0178300	AY530101	OsCPS4	3.93669	0.0179749
Os04g0180400	AK071546	CYP99A2	3.09676	0.00288931
Os04g0688200	AK103558	Peroxidase	3.3661	0.037161
Os05g0384300	AK107183	Peptidase aspartic family protein	2.37293	0.00196873
Os05g0492600	AF456247	NBS-LRR resistance-like protein	2.53676	0.0306967
Os06g0726100	AK061280	Chitinase	3.44204	0.0106932
Os06g0726200	AK061042	Chitinase	2.14672	0.0353149
Os07g0129300	AF306651	PR-1	4.53826	0.0459025
Os07g0539900	AK071889	Beta-1,3-glucanase	4.34453	1.95454E-05
Os08g0124000	AK063293	Disease resistance protein family protein	2.11057	0.00277816
Os08g0202400	AK070769	Disease resistance protein family protein	2.02296	0.0364594
Os11g0686500	AK066559	Disease resistance protein family protein	2.30764	2.04996E-05
Os12g0628600	X68197	PR-5	2.91142	0.038663
Os12g0629700	AK099946	PR-5	4.29651	0.00171269

## Discussion

### Regulation of OsWRKY53 function by the rice OsMKK4-OsMPK3/OsMPK6 cascade

We have shown here that OsWRKY53 is phosphorylated *in vitro* by the rice OsMPK3/OsMPK6 activated by OsMKK4, and that the SP cluster in the N-terminal region of OsWRKY53 is essential for the phosphorylation (Fig. 1). The SP cluster is shown to be highly conserved among several group I WRKY proteins in higher plants (Fig. S1), and also to be essential for phosphorylation of NbWRKY8 in *N. benthamiana* and AtWRKY33 in *A. thaliana* by the MEK2-SIPK/NTF4/WIPK and the AtMKK4/AtMKK5-AtMPK3/AtMPK6 cascades respectively [29], [33]. It has also been shown that the interaction of NbWRKY8 with the MAPKs depends on its D domain [29], [30]. Given that OsWRKY53 and AtWRKY33 possess the D domain as well, and the above 3 MAPKKs-MAPKs cascades from different plant species are functionally similar to one another, our findings strongly suggest that the phosphorylation mechanism of the group I WRKY proteins is well conserved in both monocots and dicots.

Coexpression of OsWRKY53 with OsMKK4^DD^, a constitutively active form of OsMKK4, increased transactivation activity of OsWRKY53 in an SP cluster-dependent manner (Fig. 3). We have also shown that OsWRKY53SD, a phospho-mimic mutant of OsWRKY53, had enhanced transactivation activity (Fig. 3), strongly suggesting that phosphorylation of OsWRKY53 within the SP cluster increases its transactivation activity. Similarly, a phospho-mimic mutant of NbWRKY8 also showed enhanced transactivation activity [29]. Given that OsWRKY53 was phosphorylated by OsMPK3/OsMPK6 activated by OsMKK4^DD^ *in vitro* (Fig. 1) and that OsMKK4^DD^ efficiently activated OsMPK3 and OsMPK6 *in vivo* [19], it is likely that OsMPK3/OsMPK6 activated by OsMKK4 phosphorylates OsWRKY53 within the SP cluster *in vivo*.

Whereas the enhanced transactivation activity of OsWRKY53SD, phosphorylation of OsWRKY53 by OsMPK6, did not alter its W-box binding activity (Fig. 2) as is the case with AtWRKY33 [33]. In contrast, phosphorylation of NbWRKY8 by MAPKs increases both W-box binding and transactivation activity [29]. In addition, phosphorylation of OsWRKY33 by OsBWMK1 enhances W-box binding activity, and coexpression of OsWRKY33 with OsBWMK1 shows enhanced transactivation activity [35]. These findings suggest that there are at least 2 different mechanisms that regulate the W-box binding activity of group I WRKY proteins even in the same plant species. The regulatory mechanisms for NbWRKY8 and OsWRKY33 DNA-binding activity via phosphorylation are still unclear; however, it has been reported that 2 VQ motif-containing proteins (SIB1 and SIB2) interact with AtWRKY33, resulting in stimulation of the W-box binding activity of AtWRKY33, and overexpression of SIB1 enhanced disease resistance to the necrotrophic pathogen *Botrytis cinerea* in an AtWRKY33-dependent manner [48]. In *Arabidopsis*, there are 34 VQ motif-containing protein genes, and expression of the majority of these is responsive to pathogen infection [49]. In the rice genome, a number of genes were found to encode VQ motif-containing proteins and our previous transcriptome data showed that some of these genes were upregulated in response to rice blast fungus infection [50]. Thus, one or more of these rice VQ motif-containing proteins may interact with OsWRKY53 to modulate W-box binding activity and contribute to disease resistance to rice blast fungus in *OsWRKY53*-overexpressing rice plants as reported previously [36]. The regulatory mechanisms for the enhanced transactivation activity of phosphorylated/phospho-mimic group I WRKY proteins remain to be elucidated. In mammals, it has been reported that phosphorylation of transcription factors by MAPKs modulates other intrinsic transcription factor activities, such as their affinities for coactivators [51], [52]. Therefore, it will be important to examine whether the enhanced transactivation activity of the phospho-mimic mutant of OsWRKY53 is regulated by interaction with coactivators.

### Overexpression of an OsWRKY53 phospho-mimic mutant alters the rice transcriptome and further enhances basal defense responses to the rice blast fungus

Overexpression of a phospho-mimic mutant of *OsWRKY53*, *OsWRKY53SD*, resulted in further-enhanced disease resistance to a virulence rice blast fungus *M. oryzae* strain Ina86–137 compared to native *W53-OX* rice plants (Fig. 4). This result implies that the observed difference in degree of disease resistance to blast fungus is correlated with the difference in transactivation activity between OsWRKY53 and OsWRKY53SD.

We performed a transcriptome analysis using NT, *W53-OX*, and *W53SD-OX* rice plants, and found that 151 out of the 280 upregulated genes in *W53SD-OX* rice plants compared to *W53-OX* plants were also upregulated in rice plants infected with the blast fungus in comparison with our previous transcriptome data. The 151 upregulated genes could be classified into 2 groups based on their expression patterns (Fig. S5). Given that OsWRKY53 is a transcriptional activator, genes in group I may be good candidates as direct targets of OsWRKY53. In fact, we found several W-box elements in promoters of most of the group I genes (Table S5).

Furthermore GO annotation analysis revealed that all the defense-related genes in the 151 upregulated genes, including beta-1, 3-glucanase, chitinase, and *PR-5*, belonged to group I (Fig. 5 and Table S5), and several of these defense-related genes were also upregulated in uninfected *W53-OX* rice plants compared to NT (Fig. S5 and Fig. 5). Thus, it is plausible that upregulation of these defense-related genes in *W53-OX* rice plants partly contributes to enhanced rice blast resistance. Most notably, these defense-related genes were found to be more upregulated in *W53SD-OX* rice plants compared to *W53-OX* plants, as were the other group I genes (Fig. 5). Therefore, it is demonstrated that overexpression of *OsWRKY53SD* causes further induction of these defense-related genes in rice plants even without blast fungus infection, resulting in the further-elevated disease resistance of *W53SD-OX* rice plants.

We also found that the group I gene, *PR-5* (Os12g0629700) was upregulated in *W53SD-OX* rice plants (Table 1). Recently, we reported that overexpression of *OsWRKY28* resulted in enhanced susceptibility to Ina86–137 and decreased accumulation of the identical *PR-5* transcripts in response to blast fungus infection [50]. Given that OsWRKY28 is a transcriptional repressor [50], these contrasting results may indicate that OsWRKY53 and OsWRKY28 act competitively to modulate the upregulated transcript levels of some defense-related genes for fine-tuning of the basal defense-response level against the rice blast fungus.

Momilactone biosynthetic genes were also upregulated in uninfected *W53SD-OX* rice plants, resulting in constitutive accumulation of momilactones only in *W53SD-OX* plants (Table 1; Fig. 6). Recently, we have shown that the *oscps4-tos17* mutant was more susceptible to rice blast fungus than was the non-transformed control, possibly due to lower levels of momilactones [53]. Therefore, it is assumed that momilactone accumulation may also be involved in the further-enhanced disease resistance of *W53SD-OX* plants to the fungus. We have also reported previously that these momilactone biosynthetic genes constitute a functional gene cluster and are regulated in a coordinate manner by a bZIP transcription factor, OsTGAP1 [46], [54]. It has also been shown that conditional expression of OsMKK4^DD^ induced biosynthesis of diterpenoid phytoalexins including momilactones. However, expression of *OsTGAP1* was not induced by OsMKK4^DD^, and OsTGAP1 was not phosphorylated by the OsMKK4-OsMPK3/OsMPK6 cascade *in vitro* [19], [55]. Given that OsWRKY53 is phosphorylated by the OsMKK4-OsMPK3/OsMPK6 cascade *in vitro*, it is likely that OsWRKY53 and OsTGAP1 are in different signaling pathways and regulate the gene cluster for momilactone biosynthesis either cooperatively or in a different manner. Interestingly, it has also been demonstrated that both AtWRKY33 and NbWRKY8 were involved in regulation of phytoalexin biosynthetic genes [29], [32], [33], also suggesting conserved roles for group I WRKY proteins in basal defense responses in higher plants.

It has been shown that *OsWRKY53* is induced by a fungal chitin elicitor [36]. Given that the chitin elicitor activated the OsMKK4-OsMPK3/OsMPK6 cascade and OsMKK4^DD^ induced the accumulation of *OsWRKY53* transcripts [19], it is likely that the OsMKK4-OsMPK3/OsMPK6 cascade activates OsWRKY53 at both the transcriptional and posttranslational levels, in response to fungal PAMPs. Our results suggest that phosphorylation of OsWRKY53 alters the rice transcriptome resulting in a further enhancement of disease resistance to the rice blast fungus, and the OsMKK4-OsMPK3/OsMPK6 cascade may play a crucial role in driving high-level activation of basal defense responses by the above dual-level regulation of OsWRKY53. Some of the defense-related genes in the group I, such as chitinases (Os01g0687400, Os06g0726100 and Os06g0726200) and *PR-5* (Os12g0629700), were also found to be upregulated by OsMKK4^DD^ in suspension-cultured rice cells [19], supporting this hypothesis. There are several group I *WRKY* genes in the rice genome that possess the SP cluster (Fig. S1). Therefore, it will be important to analyze other group I *WRKY* genes and to identify MAPKs that phosphorylate those WRKY proteins so as to provide further insights into the biological roles of group I WRKY proteins in basal defense responses to the rice blast fungus.

## Supporting Information

Figure S1 **Alignment of the deduced amino acid sequences of group I WRKYs in rice and their homologues.** The deduced amino acid sequences of *OsWRKY53/24/78/70/33/35/30*, *AtWRKY25/33*, *N. benthamiana NbWRKY8*, and *N. tabacum NtWRKY1* were aligned using the CLUSTAL W program. Highly conserved residues are shaded in black, and similar residues are shaded in gray. D domain, MAP kinase-docking domain; SP cluster, clustered serines or threonines followed by proline (SP or TP); WRKY domain, WRKY DNA-binding domain; NLS, putative nuclear localization signal.
(EPS)

Figure S2 **Overexpression of the native** ***OsWRKY53*** **and a phosphorylation-mimic mutant of** ***OsWRKY53*** **in transgenic rice plants.** qRT-PCR analysis was performed using total RNA extracted from transgenic rice plants. Values indicate relative mRNA levels normalized to the expression of the *UBQ* gene. The data are represented as mean values for at least 3 independent experiments; bars indicate the standard deviation of the mean. NT, non-transformant control rice plants; *W53-OX*, native *OsWRKY53*-overexpressing rice plants; *W53SD-OX*, phosphorylation-mimic mutant of *OsWRKY53*-overexpressing rice plants.
(EPS)

Figure S3 **Overexpression of** ***OsWRKY53*** **enhances the disease resistance of rice plants to rice blast fungus.** Ratio of the classes of lesions in transgenic rice leaves infected with rice blast fungus *Magnaporthe oryzae* Ina86–137 is shown. Washed conidia of the blast fungus were suspended in 1 mM MES-NaOH (pH 5.7) and then were inoculated on leaves of NT and *W53-OX* transgenic rice plants. The lesions were counted according to the classifications shown in Fig. 4A. Bars represent the ratio of lesions of each class to the total number of counted lesions in 3 or 4 individual leaf blades. NT, non-transformant control rice plants; *W53-OX*, native *OsWRKY53*-overexpressing rice plants.
(EPS)

Figure S4 **Lesion numbers on leaves of** ***W53-OX*** **and** ***W53SD-OX*** **transgenic rice plants infected with the rice blast.** Washed conidia of the blast fungus were suspended in 1 mM MES-NaOH (pH 5.7) and then were inoculated on leaves of *W53-OX* and *W53SD-OX* transgenic rice plants, and average numbers of lesions in the total infected area on 3 or 4 individual leaf blades of 2 independent *W53-OX* and 2 independent *W53SD-OX* transgenic rice plants infected with the rice blast are shown; *bars* indicate the standard error of the mean. Three independent experiments were performed, and a representative result is shown. There is no significant difference in the average numbers of lesions among samples by One-way ANOVA with Tukey host hoc test. *W53-OX*, native *OsWRKY53*-overexpressing rice plants; *W53SD-OX*, phosphorylation-mimic mutant of *OsWRKY53*-overexpressing rice plants.
(EPS)

Figure S5 **Expression profiles of the genes whose expressions were upregulated in the phosphorylation-mimicking mutant of** ***OsWRKY53*** **-overexpressing rice plants.** NT, OX, and SD represent non-transformed control, native *OsWRKY53*-overexpressing, and phosphorylation-mimic mutant of *OsWRKY53* (*OsWRKY53SD*)-overexpressing rice plants, respectively. Each column of NT represents the mean of 4 biological replicates, and each column of OX and SD represents the mean of 8 biological replicates. Colors represent induction (red) and repression (blue), as indicated by the color bar. The values of heat maps are relative to those in uninfected NT samples. The columns are sorted by hierarchical clustering using the Pearson correlation and average linkage methods. Mock, uninfected control; 137, *M. oryzae* Ina86–137 infection.
(EPS)

Table S1 **Plasmids used in this study.**
(DOCX)

Table S2 **Primers used for the plasmid construction.**
(DOCX)

Table S3 **Primers used for qRT-PCR analysis.**
(DOCX)

Table S4 **Primers used for quantitative genomic PCR analysis.**
(DOCX)

Table S5 **List of genes whose expression is significantly altered in** ***OsWRKY53SD*** **-overexpressing rice plants.**
(XLSX)
