# Supplementary material for: Overexpression of Phosphomimic Mutated OsWRKY53 Leads to Enhanced Blast Resistance in Rice
Source: PLoS One. 2014 Jun 3;9(6):e98737. doi: 10.1371/journal.pone.0098737 (PMC4043820; doi:10.1371/journal.pone.0098737)
Supplement: Table S1 — Plasmids used in this study. (DOCX) [file pone.0098737.s006.docx]

**Table S1. Plasmids used in this study**

| **Plasmid** | **Plasmid design and markers** | **Reference** |
| --- | --- | --- |
| pW53SA | Amp^R^, pUC18 containing 227 bp mutated *OsWRKY53* SP cluster (SA) fragment | This study |
| pW53SD | Amp^R^, pUC18 containing 227 bp mutated *OsWRKY53* SP cluster (SD) fragment | This study |
| pZErO-2 | Km^R^ | Invitrogen |
| pZE-W53SA | Km^R^, pZErO-2 containing 1.5 kb *Eco*RV/*Hind*III mutated *OsWRKY53* (*OsWRKY53SA*) fragment | This study |
| pZE-W53SA2 | Km^R^, pZErO-2 containing 1.5 kb *Sma*I/*Sal*I mutated *OsWRKY53* (*OsWRKY53SA*) fragment | This study |
| pZE-W53SD | Km^R^, pZErO-2 containing 1.5 kb *Sma*I/*Sal*I mutated *OsWRKY53* (*OsWRKY53SD*) fragment | This study |
| pZE-W53SD2 | Km^R^, pZErO-2 containing 1.5 kb *Eco*RV/*Hind*III mutated *OsWRKY53* (*OsWRKY53SD*) fragment | This study |
| pZE-W53P2.0 | Km^R^, pZErO-2 containing 2.0 kb *OsWRKY53* 5' upstream region | 1 |
| pZE-W53PmG | Km^R^, pZErO-2 containing 0.5 kb W-box-mutated *OsWRKY53* 5' upstream region | 1 |
| pET-32b(+) | Amp^R^ | Novagen |
| pET-W53 | Amp^R^, pET-32b(+) containing 1.5 kb *OsWRKY53* fragment | 2 |
| pET-W53SA | Amp^R^, pET-32b(+) containing 1.5 kb *Eco*RV/*Hin*dIII *OsWRKY53SA* fragment from pZE-W53SA cloned into *Eco*RV/*Hin*dIII sites | This study |
| pET-W53SD | Amp^R^, pET-32b(+) containing 1.5 kb *Eco*RV/*Hin*dIII *OsWRKY53SD* fragment from pZE-W53SD2 cloned into *Eco*RV/*Hin*dIII sites | This study |
| pENTR/D-TOPO | Km^R^ | Invitrogen |
| pENTR-W53 | Km^R^, pENTR/D-TOPO containing 1.5 kb *OsWRKY53* fragment | 2 |
| pENTR-W53SD | Km^R^, pENTR/D-TOPO containing 1.5 kb *OsWRKY53SD* fragment | This study |
| pENTR-MKK4DD | Km^R^, pENTR/D-TOPO containing 1.1 kb constitutive active *OsMKK4* (*OsMKK4^DD^*) fragment | 3 |
| pENTR-MPK3 | Km^R^, pENTR/D-TOPO containing 1.1 kb *OsMPK3* fragment | 3 |
| pENTR-MPK6 | Km^R^, pENTR/D-TOPO containing 1.2 kb *OsMPK6* fragment | 3 |
| pENTR-GUS | Km^R^, pENTR/D-TOPO containing 1.8 kb *GUS* fragment | Invitorogen |
| pDEST17 | Amp^R^, *T7* promoter-His-tag, Gateway vector | Invitrogen |
| pDEST17-MKK4DD | Amp^R^, pDEST17 containing 1.1 kb *OsMKK4^DD^* fragment from pENTR-MKK4DD | 3 |
| pDEST17-MPK3 | Amp^R^, pDEST17 containing 1.1 kb *OsMPK3* fragment from pENTR-MPK3 | 3 |
| pDEST17-MPK6 | Amp^R^, pDEST17 containing 1.2 kb *OsMPK6* fragment from pENTR-MPK6 | 3 |
| pT7Blue T-vector | Amp^R^ | Novagen |
| pT7-W53PW | Amp^R^, pT7Blue containing 80 bp *OsWRKY53* promoter region with W-box elements | This study |
| pT7-W53mPW | Amp^R^, pT7Blue containing 80 bp *OsWRKY53* promoter region with mutated W-box elements | This study |
| pUCAP/Ubi-NT | Amp^R^, maize *ubiquitin* promoter-*NOS* terminator | 4 |
| pUbi_RfA_Tnos | Amp^R^, pUCAP/Ubi-NT containing gateway cassette (RfA, invitorgen) cloned into *Bam*HI/*Sac*I sites after blunting | This study |
| pUbi_GUS_Tnos | Amp^R^, pUbi_RfA containing 1.8 kbp *GUS* flagment from pENTR-GUS | This study |
| pUbi_MKK4DD_Tnos | Amp^R^, pUbi_RfA containing 1.1 kb *OsMKK4^DD^* fragment from pENTR-MKK4DD | This study |
| 430T1.2 | Amp^R^, CaMV *35S* promoter-GAL4 DNA binding domain-*NOS* terminator | 5 |
| 35S-GAL4DB-W53 | Amp^R^, 430T1.2 containing 1.5 kb *OsWRKY53* fragment | 2 |
| 35S-GAL4DB-W53SA | Amp^R^, 430T1.2 containing 1.5 kb *Sma*I/*Sal*I *OsWRKY53SA* fragment from pZE-W53SA2 cloned into *Sma*I/*Sal*I sites | This study |
| 35S-GAL4DB-W53SD | Amp^R^, 430T1.2 containing 1.5 kb *Sma*I/*Sal*I *OsWRKY53SD* fragment from pZE-W53SD cloned into *Sma*I/*Sal*I sites | This study |
| GAL4-TATA-LUC-NOS | Amp^R^, GAL4 *cis*-firefly LUC-NOS terminator | 5 |
| pRL | Amp^R^, CaMV *35S* promoter-Renilla *LUC* | 5 |
| p2KG | Hm^R^, Km^R^, maize *ubiquitin* promoter, *NOS* terminator, Gateway vector | 6 |
| p2KG-W53 | Hm^R^, Km^R^, p2KG containing 1.5 kb *OsWRKY53* fragment from pENTR-W53 | This study |
| p2KG-W53SD | Hm^R^, Km^R^, p2KG containing 1.5 kb *OsWRKY53SD* fragment from pENTR-W53SD | This study |

1. Chujo *et al*., (2009) *Biosci Biotechnol Biochem* **73**:1901-1904. 4. Shimono *et al*., (2007) *Plant Cell* **19**:2064-2076.

2. Chujo *et al*., (2007) *Biochim Biophys Acta* **1769**:497-505. 5. Ohta *et al*., (2000) *Plant J* **22**:29-38.

3. Kishi-Kaboshi *et al*., (2010) *Plant J* **63**:599-612. 6. Kitagawa *et al*., (2010) *Plant Cell Physiol* **51**:1315-1329.
