# Supplementary material for: Overexpression of Phosphomimic Mutated OsWRKY53 Leads to Enhanced Blast Resistance in Rice
Source: PLoS One. 2014 Jun 3;9(6):e98737. doi: 10.1371/journal.pone.0098737 (PMC4043820; doi:10.1371/journal.pone.0098737)
Supplement: Table S2 — Primers used for the plasmid construction. (DOCX) [file pone.0098737.s007.docx]

**Table S2. Primers used for the plasmid construction**

| Primer | Sequence |
| --- | --- |
| W53-Ala F | 5’-GTGAGGAGCGCGCCCCGAGGGGGTTCTC-3’ |
| W53-Ala R | 5’-GATTGCACCGGTGGTCGGGGCCGCCAAG-3’ |
| W53-N Fw | 5’-AGAGGATATCGGCGTCCTCGACGGGGGGGTTG-3’ |
| W53-C Rv | 5’-AGAGAAGCTTCTAGCAGAGGAGCGACTCGACGAAC-3’ |
| 53 GAL4 F | 5’-AGAGCCCGGGGATGGCGTCCTCGACGGGGGGGTTG-3’ |
| 53 GAL4 R | 5’-AGAGGTCGACCTAGCAGAGGAGCGACTCGACGAAC-3’ |
| W53-Asp F | 5’-GTGAGGAGCGCGACCCGAGGGGGTTCTC-3’ |
| W53-Asp R | 5’-GATTGCACCGGTGGTCGGGTCCGCCAAG-3’ |
| 53ORF Gateway F | 5’-CACCATGGCGTCCTCGACGGGGGGGTTGGA-3’ |
| OsWRKY53 pENTR R | 5’-CTAGCAGAGGAGCGACTCGACGAAC-3’ |
| W53 Wbox Fw | 5’-GCGCGAGCCTCAGCCGCCTCGTC-3’ |
| W53 Wbox Rv | 5’-TTTGGGGATGCTTCGGCGAAG-3’ |

Restriction enzyme sites are indicated in red letters.
