# Supplementary material for: Overexpression of Phosphomimic Mutated OsWRKY53 Leads to Enhanced Blast Resistance in Rice
Source: PLoS One. 2014 Jun 3;9(6):e98737. doi: 10.1371/journal.pone.0098737 (PMC4043820; doi:10.1371/journal.pone.0098737)
Supplement: Table S3 — Primers used for qRT-PCR analysis. (DOCX) [file pone.0098737.s008.docx]

**Table S3. Primers used for qRT-PCR analysis**

| Gene Name | Locus ID | Primer sequence |
| --- | --- | --- |
| *OsWRKY53* & *OsWRKY53SD* |  | Fw: 5’-GTTCGTCGAGTCGCTCCTCTGCTAG-3’ |
|  |  | Rv: 5’-AGACCGGCAACAGGATTCAATC-3’ |
| *Ubiquitin* | Os10g0542200 | Fw: 5’-TCCGAGAGATGGGTTTCATC-3’ |
|  |  | Rv: 5’-GCCAAGATTGCCAAGAAGAC-3’ |
| *GAL4DB variants* |  | Fw: 5’-CATGCGATATTTGCCGACTT-3’ |
|  |  | Rv: 5’-AGCGGAGACCTTTTGGTTTT-3’ |
| *RLUC* |  | Fw: 5’-AACGCGGCCTCTTCTTATTT-3’ |
|  |  | Rv: 5’-ACCAGATTTGCCTGATTTGC-3’ |
| *Chitinase* | Os01g0660200 | Fw: 5’-TTGCACAGTGGTTGAGTTGG-3’ |
|  |  | Rv: 5’-GTACACCATGTGGGTTGTGC-3’ |
| *Chitinase* | Os01g0687400 | Fw: 5’-GCTACGCCTACGAACCATTC-3’ |
|  |  | Rv: 5’-GTCCGGTCGGTGTACATTCT-3’ |
| *Beta-1,3-glucanase* | Os01g0940800 | Fw: 5’-GCTGCTCATGTCAAATGCAA-3’ |
|  |  | Rv: 5’-CAAAGTGCCAAGGCAGAGTT-3’ |
| *Chitinase* | Os03g0132900 | Fw: 5’-ACGTCGTCATAAGCGGATTC-3’ |
|  |  | Rv: 5’-CGAACGCTCTCTGCTAGCTT-3’ |
| *Chitinase* | Os06g0726100 | Fw: 5’-TGATCCGAGCAGACGAATAA-3’ |
|  |  | Rv: 5’-GAAGAATGATCAAATCTTTGTTTTAGA-3’ |
| *Chitinase* | Os06g0726200 | Fw: 5’-GCTTCAAGCTTTGCACTGAT-3’ |
|  |  | Rv: 5’-CAGCCATTGTGGGCATTACT-3’ |
| *PR-1* | Os07g0129300 | Fw: 5’-GACGGCGAATCTCCCTACTA-3’ |
|  |  | Rv: 5’-AGCATGCGAACTGTGTGTGT-3’ |
| *Beta-1,3-glucanase* | Os07g0539900 | Fw: 5’-ACTCGGCAGTAAGAGGATGG-3’ |
|  |  | Rv: 5’-AAAATTCCAAACCGGAATACA-3’ |
| *PR-5* | Os12g0629700 | Fw: 5’-GCATTAGCTGGCTGCTATAGAT-3’ |
|  |  | Rv: 5’-CCATGGACGATTATTATCTTATTATTT-3’ |
| *OsCPS4* | Os04g0178300 | Fw: 5’-TGACGAGGCTGGGCATATC-3’ |
|  |  | Rv: 5’-TCTGGAGTCCAGTTCCTGAAA-3’ |
| *OsKSL4* | Os04g0179700 | Fw: 5’-GGGACTCGAGCGGTGATGT-3’ |
|  |  | Rv: 5’-TCTAGCCTCCCATCCCATGTT-3’ |
|  |  | TaqMan probe: 5’-FAM-CTGTCCCGGATATGTT-MGB-3’ |
| *OsMAS* | Os04g0179200 | Fw: 5’-AAATGATTTGGGACCAGTCG-3’ |
|  |  | Rv: 5’-GACAGAATCTAGCTAGCGATGGA-3’ |
| *CYP99A2* | Os04g0180400 | Fw: 5’-ATACGGCTCCTACCCAAAGC-3’ |
|  |  | Rv: 5’-CATTATCCGGGGACAAACAT-3’ |
| *CYP99A3* | Os04g0178400 | Fw: 5’-TCGCTTACGTGCTTGCATAC-3’ |
|  |  | Rv: 5’-CAAAGCACGGGGTATCAACT-3’ |
