# Supplementary material for: Overexpression of Phosphomimic Mutated OsWRKY53 Leads to Enhanced Blast Resistance in Rice
Source: PLoS One. 2014 Jun 3;9(6):e98737. doi: 10.1371/journal.pone.0098737 (PMC4043820; doi:10.1371/journal.pone.0098737)
Supplement: Table S4 — Primers used for quantitative genomic PCR analysis. (DOCX) [file pone.0098737.s009.docx]

**Table S4. Primers used for quantitative genomic PCR analysis**

| Gene Name | Locus ID | Primer sequence |
| --- | --- | --- |
| *M. oryzae 28S rRNA* |  | Fw: 5’-GGGGCGATTTTTAGCCTTCA-3’ |
|  |  | Rv: 5’-ATTCGAAGGCCCACGTTCAA-3’ |
| *eEF1α* | Os03g0178000 | Fw: 5’-CAACCCTGACAAGATTCCCT-3’ |
|  |  | Rv: 5’-AGTCAAGGTTGGTGGACCTC-3’ |
